# Prevalence and determinants of chronic kidney disease in women with hypertensive disorders in pregnancy in Nigeria: a cohort study

**DOI:** 10.1186/s12882-021-02419-6

**Published:** 2021-06-18

**Authors:** Salisu M. Ishaku, Timothy Olusegun Olanrewaju, Joyce L. Browne, Kerstin Klipstein-Grobusch, Gbenga A. Kayode, Arie Franx, Diederick E. Grobbee, Charlotte E. Warren

**Affiliations:** 1 Julius Global Health, Julius Center for Health Sciences and Primary Care, University Medical Center Utrecht, Utrecht University, Utrecht, The Netherlands; 2 Division of Nephrology, Department of Medicine, University of Ilorin and University of Ilorin Teaching Hospital, Ilorin, Nigeria; 3 Division of Epidemiology and Biostatistics, School of Public Health, Faculty of Health Sciences, University of the Witwatersrand, Johannesburg, South Africa; 4 Translational Health Sciences, Bristol Medical School, University of Bristol, Southmead Hospital, Bristol, UK; 5 Erasmus Medical Center, University Medical Center Rotterdam, Rotterdam, The Netherlands; 6 Population Council, Washington DC, USA

**Keywords:** Hypertensive disorder in pregnancy, Chronic kidney disease, Nigeria, Low- and middle-income countries, Sub-Saharan Africa

## Abstract

**Background:**

Worldwide, hypertensive disorders in pregnancy (HDPs) complicate between 5 and 10% of pregnancies. Sub-Saharan Africa (SSA) is disproportionately affected by a high burden of HDPs and chronic kidney disease (CKD). Despite mounting evidence associating HDPs with the development of CKD, data from SSA are scarce.

**Methods:**

Women with HDPs (*n* = 410) and normotensive women (*n* = 78) were recruited at delivery and prospectively followed-up at 9 weeks, 6 months and 1 year postpartum. Serum creatinine was measured at all time points and the estimated glomerular filtration rates (eGFR) using CKD-Epidemiology equation determined. CKD was defined as decreased eGFR< 60 mL/min/1.73m^2^ lasting for ≥ 3 months. Prevalence of CKD at 6 months and 1 year after delivery was estimated. Logistic regression analyses were conducted to evaluate risk factors for CKD at 6 months and 1 year postpartum.

**Results:**

Within 24 h of delivery, 9 weeks, and 6 months postpartum, women with HDPs were more likely to have a decreased eGFR compared to normotensive women (12, 5.7, 4.3% versus 0, 2 and 2.4%, respectively). The prevalence of CKD in HDPs at 6 months and 1 year postpartum was 6.1 and 7.6%, respectively, as opposed to zero prevalence in the normotensive women for the corresponding periods. Proportions of decreased eGFR varied with HDP sub-types and intervening postpartum time since delivery, with pre-eclampsia/eclampsia showing higher prevalence than chronic and gestational hypertension. Only maternal age was independently shown to be a risk factor for decreased eGFR at 6 months postpartum (aOR = 1.18/year; 95%CI 1.04–1.34).

**Conclusion:**

Prior HDP was associated with risk of future CKD, with prior HDPs being more likely to experience evidence of CKD over periods of postpartum follow-up. Routine screening of women following HDP-complicated pregnancies should be part of a postpartum monitoring program to identify women at higher risk. Future research should report on both the eGFR and total urinary albumin excretion to enable detection of women at risk of future deterioration of renal function.

**Supplementary Information:**

The online version contains supplementary material available at 10.1186/s12882-021-02419-6.

## Introduction

Hypertensive disorders in pregnancy (HDPs) complicate 5–10% of pregnancies world-wide [1, 2] and are annually responsible for more than 500,000 and 70,000 global neonatal and maternal deaths respectively [2]. HDPs include chronic hypertension, gestational hypertension and (pre-) eclampsia [1, 2]. Besides their contribution to immediate pregnancy-related complications, HDPs are associated with long-term health consequences for both the mothers (cardiovascular diseases, type II diabetes, kidney diseases and mental health problems) and their babies (prematurity and low birth weight) [1, 2].

The association between kidney disease and HDPs (especially pre-eclampsia) [3] could be complex due to possibility of reverse causality. On one hand, pre-existing but undiagnosed renal diseases can be unmasked by pregnancy (manifesting as hypertension and or proteinuria) [4] leading to spurious diagnosis of HDPs [5]. On the other hand, renal function abnormalities can occur de novo in association with HDPs, especially pre-eclampsia [3]. In both situations, the renal impairments may persist, and could lead to chronic kidney diseases (CKD) [3]. In addition, pre-eclampsia affects renal morphology [6], leads to persistent microalbuminuria [7–9], and progression of CKD to end-stage kidney disease (ESKD) [10–13]. The associations between pre-eclampsia and CKD were shown to be stronger within 5 years of index pregnancies and weaker thereafter [6, 11].

Kidney disease is defined as “an abnormality of kidney structure or function with implications for the health of an individual, which can occur abruptly, and may either resolve or become chronic” [14]. When kidney disease is present for ≥3 months, it is described as “chronic kidney disease (CKD)” [14]. In 2017, CKD accounted for 1.2 million and 35.8 million, respectively, of global deaths and disability adjusted life years (DALYs), with sub-Saharan Africa experiencing heavier burden for its state of development [15]. In 2017, 13,740 deaths occurred from kidney disease in Nigeria [15]. Apart from deaths and disabilities from CKD, renal replacement therapies can be very prohibitive in many LMICs. A systematic review estimated that annual costs of hemodialysis and peritoneal dialysis in LMICs ranged, respectively, from $3424 to $42,785 and $7974 to $47,971 [16]. This suggests the importance of early screening of CKD following HDPs.

The contribution of HDPs to the burden of CKD is not fully elucidated in sub-Saharan Africa in general and Nigeria in particular. Scarce data from a cross sectional study undertaken during pregnancy in Nigeria showed an association between pre-eclampsia and markers of kidney dysfunction [17] and a small short-term follow up study of Cameroonian women with severe pre-eclampsia revealed persistent proteinuria of 31.5 and 1.8% at 3 and 6 months postpartum, respectively [18]. To date, no study from sub-Saharan Africa prospectively evaluated prevalence of CKD following deliveries of pregnancies complicated by hypertensive disorders. In this study, we prospectively followed cohorts of women with HDPs and normotensive pregnancies to examine the prevalence and determinants of CKD over a 1-year period after delivery, at tertiary care settings in Nigeria.

## Methodology

### Study design

The study was a prospective cohort study. Women with HDP and women with normotensive pregnancies who delivered in the participating hospitals were recruited from August 2017 to April 2018 and followed up over a subsequent period of 1 year. The last woman recruited exited the study on March 31, 2019 after 1 year of follow up.

### Study setting

The study was conducted at eight tertiary hospitals in the six geo-political zones of Nigeria. The hospitals were purposefully selected to reflect diversity in the country in terms of ethnic differences and socio-economic status. The following states (and hospitals) participated: Bauchi State (Abubakar Tafawa Balewa University Teaching Hospital, ATBUTH), Cross River State (University of Calabar Teaching Hospital, UCTH), Ebonyi State (Federal Teaching Hospital Abakaliki, FTHA), Kogi State (Federal Medical Center, FMC Lokoja), Kano State (Aminu Kano Teaching Hospital, AKTH), Ondo State (Mother and Child Hospital Akure and University of Medical Sciences Teaching Hospital, Ondo) and Sokoto State (Usmanu Danfodio University Teaching Hospital, UDUTH). The facilities were high-volume sites with well-functioning antepartum, intrapartum, and postpartum clinics, delivery rooms, and laboratory services with combined average annual deliveries of 38,400.

### Participants

Receiving delivery care services at the facilities, being 18 years or above, and a diagnosis of HDPs were the main eligibility criteria for inclusion in the study. The unmatched normotensive women were recruited from the same hospitals. HDP and normotensive women were recruited from similar population of women giving birth in these facilities. Recruitment proceeded independently at all facilities. Case identification was carried out by specifically trained and experienced midwives using standard diagnostic criteria based on HDP definitions described above. Women with the following conditions (based on previously documented clinical history and diagnoses) were excluded: having multiple pregnancies, diabetes mellitus, sickle cell disease, heart disease, preexisting kidney disease and connective tissues disorders.

### Study procedures

Normotensive women and women with HDPs were informed of the study either during antenatal care or after delivery. All recruitment took place in the postpartum period. Those willing to participate were individually counseled and signed or thumb-printed an informed consent form (consent rate over 95% among HDPs, 35% among normotensive). Enrollment forms, collecting information on socio-demographic and obstetrics variables such as age, body mass index (BMI), parity and booking status, were completed within 24 h of delivery. After enrollment, the women underwent general and systemic clinical examination. In addition, laboratory investigations, including urine protein analysis and renal function tests, were performed on the participants before they were discharged from the hospitals. They were subsequently followed up undergoing the same clinical and laboratory investigations conducted at baseline, at 9 weeks, 6 months and 1 year postpartum. To improve follow up rate, the research participants were requested to provide their contact information and spousal mobile telephone numbers. They were reminded of their follow up appointments through phone calls. Participants’ contact information was not linked to their clinical records while all clinical information was linked to unique identifiers.

### Exposure variables

The main exposures of interest were the presence of any of the HDP sub-type including chronic hypertension, gestational hypertension and pre-eclampsia as defined by the International Society for the Study of Hypertension in Pregnancy – ISSHP - (all cases of chronic hypertension with super-imposed pre-eclampsia were classified simply as pre-eclampsia) [1, 2]. Hypertension was defined as systolic blood pressure of ≥140 mmHg and or diastolic blood pressure of ≥90 mmHg measured on two consecutive periods 4–6 h apart. Chronic hypertension in pregnancy was defined as any hypertension with onset before the index pregnancy or diagnosed within the first 20 weeks of the index pregnancy. Gestational hypertension was defined as any hypertension occurring after the first 20 weeks of pregnancy without significant proteinuria (< 2++ of proteinuria on urine dipstick measurement) or any hematological or biochemical abnormality. Pre-eclampsia was defined as hypertension with onset after the first 20 weeks of pregnancy with significant proteinuria (≥2++ of proteinuria on urine dipstick measurement) or the presence of any hematological and biochemical abnormality [1, 2].

### Outcome variables

We assessed prevalence of CKD based on serum creatinine (Modified Jaffe Kinetic Method - Roche C311 and Abbott C4000) and estimated glomerular filtration rate (eGFR) by CKD-Epidemiology equation as recommended by the Kidney Disease Improving Global Outcomes for black women [19]:

- eGFR for creatinine ≤0.7 mg/dl: 144 * (serum creatinine/0.7)^-0.239^ * 0.993^Age^ *1.159]
- eGFR for creatinine > 0.7 mg/dl: 144 * (serum creatinine/0.7)^-1.209^* 0.993^Age^ *1.159]

The CKD-epidemiology equation was used together with the GFR calculator of the National Kidney Foundation available at https://www.kidney.org/professionals/kdoqi/gfr_calculator. When the eGFR was < 60 mL/min/1.73m^2^, it is considered decreased and when decreased eGFR persists for ≥ 3 months, it indicates presence of CKD [6, 15].

### Data source/data collection

At baseline-within 24 h of delivery, 9 weeks, 6 months and 1 year postpartum, laboratory tests were performed on the women. We collected blood samples for serum urea and creatinine measurement and estimated glomerular filtration rate. Clinical examination, blood and urine sample collection were performed by trained medical officers who served as research assistants for this study. Laboratory tests were performed using techniques as reported above. Both the medical officers and the laboratory scientists were not aware of clients’ categorization as either HDPs or normotensive.

### Sample size

Based on previous evidence that about 14% of women with pre-eclampsia had abnormal GFR, at least, 4 months after delivery [18], we estimated that 185 women would be required to participate in the study, each for HDPs and normotensive (at 5% alpha level and power of 80%). Considering 10% potential loss to follow-up, 204 women were required to be recruited in each arm. However, we planned to enroll as many women as resources could accommodate given that the study was observational with minimal risks to the women. Because of the differential consenting rates between the two cohorts (95 and 38% for HDPs and normotensive respectively – due to low perception of risk and threat among the normotensive), we were able to recruit 410 and 78 women with HDPs and normotensive pregnancies respectively.

### Data management and statistical analysis

The results of medical and laboratory investigations were entered into electronic data capturing platform (Open data kit - ODK) by trained research assistants. All women with HDPs and normotensive women who were recruited and successfully followed-up for each period were analyzed using SPSS IBM version 25.0. Frequencies, percentages, proportion and means (standard deviations) were used to describe participants’ baseline characteristics. For comparison of mean eGFR between normotensive women and women with HDP, independent t-tests was used in case of normally distributed data, otherwise the Mann–Whitney U test was applied. To compare mean differences in eGFR between HDP sub-types (gestational hypertension, pre-eclampsia and eclampsia), one-way analysis of variance (ANOVA) was performed. A Tukey post hoc test was used to determine where the difference lied between the HDP sub-types in their mean eGFR. Univariable and multivariable logistic regression analyses were performed to assess risk factors for CKD at 6 months and 1 year postpartum. Confounders’ selection was theory-driven, and the following variables were included: age, BMI, parity, gestational age at delivery, early- and late-onset HDP, booking status and HDP sub-types. All the included variables were taken as numerical in the logistic regression analyses.

### Loss to follow up

Cases of loss to follow up were nearly similar between HDP and normotensive women and occurred when subjects failed to report for data collection for each period. Of the 410 women with HDPs enrolled, 147(36%), 178(43%) and 132(32%) were lost to follow up at 9 weeks, six months and 1 year postpartum, respectively. The corresponding values for the normotensive women were 25(32%), 35(45%) and 19(24%) respectively. We assumed data were missing completely at random. Therefore, complete case analysis was performed such that for any data collection period only clients that have reported and provided complete information were analyzed.

### Ethical approval

The study was approved by the Population Council’s institutional review board in New York (protocol no. 810), National Health Research Ethics Committee (NHREC) at the Federal Ministry of Health of Nigeria and by the institutional review boards at all the participating hospitals.

## Results

**Fig. 1:**
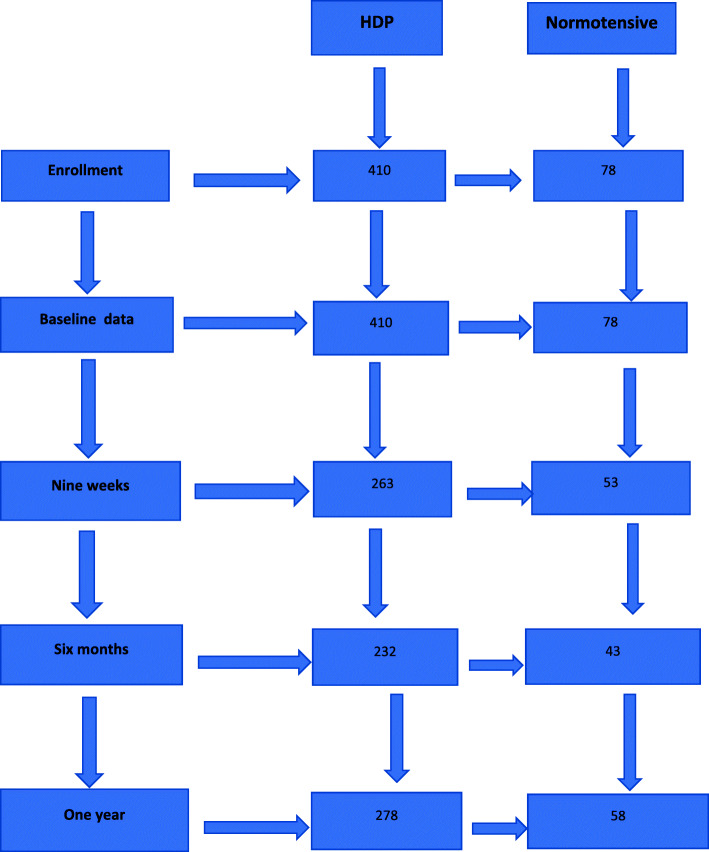
Follow-up rates among women with HDP and normotensive pregnancies

Figure 1 shows flowchart of follow up of women with HDPs versus the normotensive over the one-year period. The number of women assessed at various time points varied due to missed appointment: 263 (65%), 232 (57%), 278 (68%) and 53 (68%), 43 (55%), and 58 (74%) for HDPs and control at 9 weeks, 6 months and 1 year, respectively.

Table 1 describes the baseline characteristics of women with HDPs and those with normotensive pregnancies. A total of 410 women with HDPs and 78 with normotensive pregnancies were enrolled. Women with HDPs and normotensive were not significantly different in respect to their mean age [29.5(Sd = 13.8) versus 28.3(Sd = 5.1), *p* = 0.48] and BMI [28.9(Sd = 7.7) versus 26.8(Sd = 6.4), *p* = 0.05]. The proportion of multiparity (≥4 previous deliveries) was similar in the two groups (24%). Similarly, there was no significant difference in gestational age at booking [with mean booking gestational age of 23.7 weeks (Sd = 6.3) and 22.8 weeks (Sd = 6.4), *p* = 0.301] for women with HDPs and normotensive, respectively. Of women with HDPs, about two-thirds (62.3%) were late-onset (≥ 34 weeks of gestation). However, based on HDP sub-types, late-onset disease occurred in 85, 74 and 61% of women with gestational hypertension, eclampsia, and pre-eclampsia respectively. The prevalence of CKD in HDPs at 6 months and 1 year postpartum was 6.1 and 7.6%, respectively, as opposed to zero prevalence in the normotensive cohort for the corresponding periods.

**Table 1:** Baseline characteristics of 410 women with hypertensive disorders in pregnancy and 78 women with normotensive pregnancy

Variables	HDPs n(%) m [SD]	Normotensive n (%) m [SD]	*P*-value	GHT (***N*** = 73)	CHT (***N*** = 33)	PE (***N*** = 200)	EC (***N*** = 58)
Age, Mean (SD)	29.5[13.8]	28.3[5.1]	0.48	35.2(6.8)	33.3(28.9)	28.4(5.7)	24.6(6.3)
Booking BMI, Mean (SD)	28.9[7.7]	26.8[6.4]	0.05	31.6(8.5)	31.7(11.7)	27.7(6.2)	24.9(4.2)
*Parity*							
Para 0	86(21)	10(12.8)		9(11.5)	1(1.3)	45(57.7)	23(29.5)
Para 1–3	226(55)	49(62.8)		51(25.5)	14(7.0)	108(54.0)	27(13.5)
≥ para 4	98(24)	19(24.4)		15(17.24)	18(20.69)	47(54.02)	7(8.05)
*Booking status*							
Booked	247(60.2)	66(89.7)		60(80)	23(70)	117(58.5)	21(36.8)
Unbooked	163(39.8)	8(10.3)		15(20)	10(30)	83(41.5)	36(63.2)
Booking GA, Mean (SD)	23.7[6.3]	22.8[6.4]	0.301	24.8(6.7)	23.8(4.6)	23.1(6.3)	23.0(6.7)
*GA at booking*							
≤ 12 weeks	14 (3.4)	3 (3.8)		3(4.0)	0(0.0)	7(3.5)	2(3.5)
13–20 weeks	73 (17.8)	23 (29.5)		14(18.7)	6(18.2)	43(21.5)	6(10.3)
> 20 weeks	323 (78.8)	52 (66.7)		58(77.3)	27(81.8)	150(75.0)	50(86.2)
GA at HDP onset, Mean (SD)	33.2[8.7]	–		36.9(4.1)	23.8(13.4)	33.0(8.5)	34.5(6.5)
*GA of onset of HDP*							
≥ 34 weeks	225(62.3)	–		61(84.7)	0(0.0)	122(61.3)	42(73.7)
< 34 weeks	136(37.7)	–		11(15.3)	33(100)	77(38.7)	15(26.3)
Delivery GA, Mean (SD)	36.5[4.1]	38.3[1.7]	0.301	38.7(1.9)	36.1(3.9)	35.9(4.4)	35.5(4.3)
*Perinatal deaths n (%)*							
Stillbirths	29 (7.1)	0.0 (0.0)		2(6.9)	1(3.4)	20(69.0)	6(20.7)
Early neonatal deaths	12 (2.9)	1.0 (1.3)		1(8.3)	2(16.7)	9(75.0)	0(0.0)

*HDPs* Hypertensive disorders in pregnancy, *SD* standard deviation, *GHT* Gestational hypertension, *CHT* Chronic hypertension, *PE* Pre-eclampsia, *EC* Eclampsia, *GA* gestational age

Table 2 shows mean differences in eGFR between women with HDPs and those with normotensive pregnancies over 1 year since delivery. eGFR was significantly higher in normotensive cohort at baseline (mean difference = 11.88 mL/min/1.73m^2^, *p* = 0.015) and at 1 year after delivery (mean difference = 8.76 mL/min/1.73m^2^, *p* = 0.039).

**Table 2:** Comparison of mean and proportions with estimated glomerular filtration rates (eGFR) between women with hypertensive disorders in pregnancy and the normotensive over one postpartum year

Timeline	HDPs, Mean (sd)	Normotensive, Mean (sd)	Mean difference	***p***-values	Proportions with decreased eGFR < 60 mL/min/1.73m^**2**^	
					HDPs	Normotensive
Baseline	103.52(39.8)	115.40(28.8)	11.88	0.015	49/408 = 12.0%	0/73 = 0.0%
9 weeks	108.19(31.7)	112.69(26.3)	4.50	0.339	15/263 = 5.7%	1/50 = 2.0%
6 months	106.33(29.0)	113.64(27.9)	7.31	0.136	10 /233 = 4.3%	1/41 = 2.4%
1 year	103.99(28.3)	112.75(30.7)	8.76	0.039	10/281 = 3.5%	3/55 = 5.5%

At baseline (within 24 h of delivery), 9 weeks and 6 months postpartum, there were higher proportions of women with decreased eGFR in HDP cohort as compared to their normotensive counterparts (12, 5.7, 4.3% versus 0, 2 and 2.4% respectively – see Table 2). The prevalence of decreased eGFR varies with HDP sub-type and the length of time since delivery. At baseline, it was 17.9, 14.7, 9.1 and 6.7% in women with eclampsia, pre-eclampsia, chronic and gestational hypertension respectively. At 1 year after delivery, chronic hypertension and gestational hypertension had relatively higher proportions of decreased eGFR (0, 3.8, 8.3 and 3.6% for eclampsia, pre-eclampsia, chronic hypertension, and gestational hypertension, respectively).

**Fig. 2:**
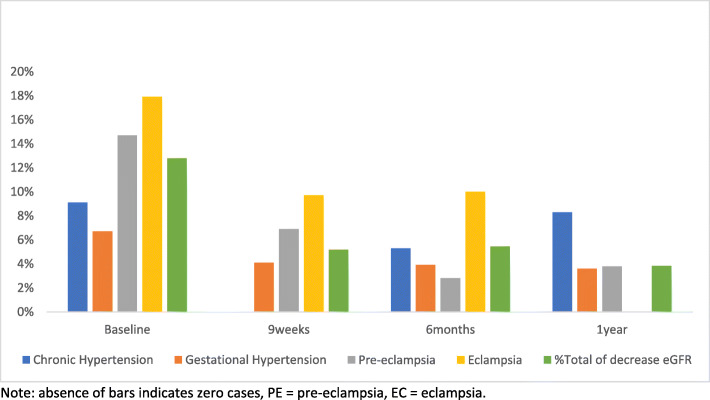
Percentage of women with eGFR < 60 mL/min/1.73m^2^ among the various HDP categories from baseline (within 24 h of delivery) up to one year

Figure 2 shows changing prevalence of eGFR among the HDP categories from delivery until 1 year thereafter, and Table 3 shows analysis of variance of mean differences in eGFR between HDP sub-types (chronic hypertension, gestational hypertension, pre-eclampsia/eclampsia) over 1 year since delivery. Significant differences in mean eGFR existed between the HDP sub-types at baseline (*p* = 0.0015) and at 6 months after delivery (*p* = 0.0194). Based on post-hoc test, the difference at baseline was between women with eclampsia and gestational hypertension (*p* = 0.001) while at 6 months after delivery, the difference was between pre-eclampsia and chronic hypertension (*p* = 0.014).

**Table 3:** Analysis of variance of mean estimated glomerular filtration rate between HDP-types (chronic hypertension, gestational hypertension, pre-eclampsia and eclampsia) over one postpartum year

HDP Categories	N (%)	Mean (SD)	***P***-value
**Baseline**			
Chronic hypertension	33 (9.11)	102.89(37.9)	0.002
Gestational Hypertension	75 (20.71)	112.59(31.12)	
Pre-eclampsia	198 (54.69)	99.55(34.45)	
Eclampsia	56 (15.47)	89.23(34.57)	
**Total**	**362**		
**9 weeks**			
Chronic hypertension	21 (8.36)	109.98(25.9)	0.653
Gestational Hypertension	49 (19.52)	110(31.91)	
Pre-eclampsia	131 (52.19)	105.94(31.94)	
Eclampsia	31 (12.35)	102.48(33.69)	
**Total**	**251**		
**6 months**			
Chronic hypertension	19(9.18)	87.39(27.94)	0.02
Gestational Hypertension	51(24.63)	105.92(29.12)	
Pre-eclampsia	107(51.69)	109.43(29.76)	
Eclampsia	30(14.49)	101.49(24.58)	
**Total**	**207**		
**1 Year**			
Chronic hypertension	24(9.71)	94.83(26.27)	0.086
Gestational Hypertension	56(22.67)	107.67(30.04)	
Pre-eclampsia	133(58.85)	105.34(28.55)	
Eclampsia	34(13.77)	95.70(24.73)	
**Total**	**247**		

At 1 year postpartum, while the proportion of women with decreased eGFR was higher in the normotensive women than in the HDP cohort (5.5 and 3.5% respectively), the mean creatinine value was higher in the HDP cohort [(945umol/L (Sd = 570) versus 844umol/L (Sd = 270)].

Finally, Table 4 describes the results of univariable and multivariable logistic regression analyses of risk factors for decreased eGFR in women with HDPs at 6 months and 1 year after delivery. Only maternal age independently shown to be significant risk for decreased eGFR at 6 months postpartum (aOR = 1.18/year; 95%CI = 1.03–1.36). At 1 year after delivery, none of the included variables was a significant risk factor.

**Table 4:** Univariable and Multivariable logistic regression of demographic and obstetrics factors and decreased eGFR (60 mL/min/1.73m^2^) in women with hypertensive disorders in pregnancy at 6 months and 1 year postpartum

	6 months		1 year	
	***Univariable analysis***	***Multivariable analysis***	***Univariable analysis***	***Multivariable analysis***
**Variables**	***OR (95%CI;p-value)***	***OR (95%CI; p-value)***	***OR (95%CI; p-value)***	***OR (95%CI; p-value)***
Age	1.09(0.99–1.20;0.06)	1.18(1.03–1.36;0.02)	1.06(0.98–1.17;0.13)	1.03(0.91–1.16;0.60)
Parity	0.67(0.25–1.81;0.43)	0.33(0.07–1.58;0.17)	1.08(0.46–2.54;0.85)	1.07(0.32–3.60;0.90)
BMI	1.01(0.93–1.11;0.81)	0.99(0.88–1.13;0.94)	1.01(0.94–1.09;0.79)	0.97(0.89–1.06;0.54)
GA at delivery	1.09(0.86–1.38;0.47)	1.25(0.83–1.89;0.29)	0.96(0.85–1.08;0.51)	0.96(0.84–1.11;0.59)
Early-onset HDP	***	***	***	***
Late-onset HDP**	1.00(0.24–4.13;1.00)	0.79(0.062–10.31;0.29)	0.69(0.18–2.51;0.57)	1.02(0.18–5.73;0.90)
Booking status**	0.99(0.28–3.47;0.99)	1.41(0.19–10.06;0.73)	5.67(0.73–44.21;0.09)	4.04(0.48–34.2;0.20)
Chronic HTN	***	***	***	***
Gestational HTN**	0.74(0.63–8.60;0.81)	0.81(0.02–40.5;0.92)	0.41(0.54–3.8;0.38)	0.48(0.044–5.39;0.56)
Pre-eclampsia**	0.52(0.05–5.27;0.57)	1.45(0.05–43.5;0.83)	0.43(0.78–2.35;0.33)	0.52(0.07–4.08;0.54)
Eclampsia**	2.00(0.19–20.7;0.56)	3.71(0.04–354.6;0.57)	***	***

^***^Omitted variables due to small number of observations, ^**^categorical variable taken as numerical variables

## Discussion

The 2012 Kidney diseases: Improving Global Outcomes (KDIGO) provides guiding criteria for identification of chronic kidney disease which include either determining the presence of one or more markers of kidney disease or identifying presence of decreased estimated GFR (eGFR) (< 60 mL/min/1.73m^2^) lasting ≥ 3 months [14, 19]. We used eGFR derived from serum creatinine (eGFR_creat_) to assess CKD in this study because it is the recommended approach [19]. We, therefore, reported prevalence of both the decreased eGFR and CKD. The findings showed that, at delivery, none of the normotensive women had decreased eGFR as opposed to 12% prevalence among the HDPs is suggestive of adverse effect that HDP could have on renal function, with pre-eclampsia or eclampsia having more profound renal effect than chronic or gestational hypertension. To the best knowledge of the authors, no previous study reported eGFR at delivery following HDPs.

Based on KDIGO guideline (2012), 7.6% of HDPs women with CKD at 1 year postpartum in our study will require yearly monitoring of their renal function [19], as opposed to 13.7% reported for Dutch women [18]. We expect the prevalence to be higher in the Dutch study because the measurement was exclusively performed among women with pre-eclampsia who have the highest risk [10–13]. This is comparable to our pre-eclampsia participants with decreased eGFR at 6 months after delivery (13.7% versus 12.8% respectively) [20]. It is not unexpected that pre-eclampsia/eclampsia are associated with more profound renal impairment than the other HDP sub-types as they result in glomerulo-endotheliosis which affects renal parenchyma and functions, an effect absence in chronic and gestational hypertension [21–23]. However, a Taiwanese study reported that previous history of any HDP type has greater than 9 folds risk of leading to future CKD (HR = 9.38 (95% CI 7.09–12.4) [10]. A long-term Scottish cohort study revealed prevalence of CKD of 7.5 and 5.2% in women who previously had gestational hypertension and pre-eclampsia, respectively [24].

As pregnancy-related effect of HDPs on kidney function wanes in the postpartum period, the proportions of women with decreased eGFR among the HDPs declined progressively up to 6 months. However, at every postpartum time, mean eGFR is lower in HDPs than in their normotensive counterpart. This pattern was also observed by Paauw et al. in Groningen, although this was from a historical cohort after several years since delivery [25]. But at the same time, proportions of decreased eGFR rose progressively (albeit marginally) among the normotensive women. It could mean that while HDPs adversely affects renal function in pregnancy resulting in decreased eGFR, women who remain normotensive during pregnancy may not show renal function derangement until after delivery perhaps in those with pre-existing renal impairments. This suggestion requires further investigation.

HDP categories do not seem to affect kidney function to the same degrees, and their differential impact seemed to vary with increasing length of postpartum follow up time. Immediately after delivery and up to the end of puerperium, kidney function was better in descending order of gestational hypertension, chronic hypertension, pre-eclampsia, and eclampsia based on the eGFR. After six postpartum months onward, women with chronic and gestational hypertension in pregnancy fared the worst, although pre-eclampsia continued to be associated with decreased eGFR. While it is understood that pre-eclampsia affects kidney function more than the other HDP sub-types, why women with gestational/chronic hypertension had better eGFR than pre-eclampsia/eclampsia in first 6 months of delivery with reversing trend thereafter is not very clear and has not being reported previously.

Given the strong association between maternal age and CKD among our HDP cohort, the importance of maternal age in the management of HDP becomes necessary. End-stage renal disease occurred in Norwegian women with previous pre-eclampsia at mean age of 41 years [12]. Women in sub-Saharan Africa have relatively wider reproductive experience (commencing childbirth much earlier and stopping later) than elsewhere, with mean age of women seeking to limit pregnancy and childbirth being 37 years [26]. In this study, women as old as 57 were still giving birth. This implies that women in the sub-region could suffer double jeopardy in respect to HDP on account of their relatively advanced maternal age; have higher predisposition to HDP which increases the likelihood of future chronic health conditions including chronic kidney diseases.

### Study strength and limitations

Globally, this is among the first studies to estimate the prevalence of chronic kidney disease based on estimated GFR as recommended by the KDIGO 2012, and to the best of authors’ knowledge the first in sub-Saharan Africa especially in women with previous history of HDPs. Because our cohort was recruited and assessed at delivery and prospectively followed for 1 year, we were able to report prevalence of decreased eGFR within 24 h after childbirth. This information is lacking in previous studies. Our study is not without limitations. While we aimed to recruit a minimum of 185 normotensive pregnancies, we only recruited less than half as a results of lower consent rates by women with normal pregnancies. This could have led to insufficient power to detect presence of decreased eGFR and or CKD as appropriate among the normotensive cohort. On the other hand, our sample size is sufficient to suggest that these will be at the very low ends and still justifies our conclusion to focus efforts on reducing CKD prevalence primarily among women with HDP who are at highest risk.

Although our study was considerably affected by high number of missed appointments at various follow-up periods, the proportions of missed appointments were nearly similar at all time periods between women with HDPs and those with normotensive pregnancies (35% vs 32, 43% vs 45 and 32% vs 26% missed appointment rates at 9 weeks, 6 months and 1 year respectively). Because our assumption to data missing completely at random may not hold true, the complete case analysis performed could have led to erroneous estimates. However, analyses of loss to, versus complete follow up at 1 year for both the HDPs and the normotensive did not show significant difference in most of the demographic and clinical parameters between the two groups (see supplement Table I & II). Only among the HDP cohort that serum creatinine and cholesterol were significantly different with loss to follow up having higher mean creatinine values at baseline. This means that the prevalence of CKD reported in this article could have been higher were the loss to follow ups accounted for. Serum cholesterol is not known to affect renal functions.

We were unable to report on the risk of deteriorating kidney function because to do this requires estimating both the GFR and a 24-h urinary albumin excretion which we could not do under this research setting. Although some investigators reported that risk of kidney diseases following HDPs becomes weaker after 5 years of delivery [6, 11], others reported an average of 17 years postpartum for kidney disease occurrence after pre-eclampsia in first pregnancy [12]. We were unable to follow our cohorts for these durations which limits our ability to comment on long-term association between HDPs and CKD. Finally, we have no information on women’s pre-existing medical conditions which might have confounded our observations and interpretation by ascribing renal impairment to prior HDP in women with undocumented pre-existing renal disease before pregnancy, as reported by other researchers [9].

## Conclusion

Due to high fertility rate in sub-Saharan Africa, HDPs will continue to place a high burden on women’s health during their reproductive lives and beyond. With the mounting evidence suggesting an association between prior HDPs and risk of future CKD, routine screening of women following HDP-complicated pregnancies should be part of our postpartum workup to identify women in need of close monitoring. In addition, future studies should report both the eGFR and urinary albumin excretion to detect women at risk of deteriorating renal function. As well as improving renal health outcomes for women with HDPs, early detection and management of CKD will prevent the huge financial burden associated with renal replacement therapies.

### Guidelines

The authors confirm that all methods were carried out in accordance with relevant guidelines and regulations.

### Informed consent

Informed consent was taken by all the participants. Those who were less than 18 are considered emancipated minors in Nigeria by virtue of their pregnancy status and thus eligible to provide informed consent.

## Supplementary Information

**Additional file 1: Supplement I.** Comparative analyses of characteristics of completed versus loss to follow up at one year among women with hypertensive disorders in pregnancy and the normotensive counterpart. **SUPPLEMENT II.** Distribution of Sociodemographic and Obstetric Characteristic of completed versus lost to follow up among the HPDs at one year.

## Data Availability

The datasets used and/or analyzed during the current study are available from the corresponding author on reasonable request.

## Abbreviations

HDPs: Hypertensive disorders in pregnancy
CKD: Chronic Kidney disease
ESKD: End-stage kidney disease
DALYs: Disability-adjusted life years
ATBUTH: Abubakar Tafawa Balewa university teaching hospital
UCTH: University of Calabar teaching hospital
FTHA: Federal teaching hospital Abakaliki
FMC: Federal medical center
AKTH: Aminu Kano teaching hospital
UDUTH: Usmanu DanFodio university teaching hospital
BMI: Body mass index
eGFR: Estimated glomerular filtration rate
ODK: Open data kit
ANOVA: Analysis of variance
NHREC: National health research ethics committee
KDIGO: Kidney disease improving global outcomes
USAID: United States Agency for International Development
