# Supplementary material for: Prevalence and determinants of chronic kidney disease in women with hypertensive disorders in pregnancy in Nigeria: a cohort study
Source: BMC Nephrol. 2021 Jun 18;22:229. doi: 10.1186/s12882-021-02419-6 (PMC8212529; doi:10.1186/s12882-021-02419-6)
Supplement: Supplementary file 1 — Additional file 1: Supplement I. Comparative analyses of characteristics of completed versus loss to follow up at one year among women with hypertensive disorders in pregnancy and the normotensive counterpart. SUPPLEMENT II. Distribution of Sociodemographic and Obstetric Characteristic of completed versus lost to follow up among the HPDs at one year. [file 12882_2021_2419_MOESM1_ESM.docx]

**Prevalence and determinants of chronic kidney disease in women with hypertensive disorders in pregnancy in Nigeria: a cohort study**

Salisu M. Ishaku^1^, Timothy Olusegun Olanrewaju^1,2^, Joyce L. Browne^1^, Kerstin Klipstein-Grobusch^1,3^, Kayode Gbenga^4^, Arie Franx^5^, Diederick E. Grobbee^1^, Charlotte Warren^6^,

**Affiliations**

^1^Julius Global Health, Julius Center for Health Sciences and Primary Care, University Medical Center Utrecht, Utrecht University, The Netherlands

^2^University of Ilorin Teaching Hospital, Ilorin, Kwara State, Nigeria

^3^ Division of Epidemiology and Biostatistics, School of Public Health, Faculty of Health Sciences, University of the Witwatersrand, Johannesburg, South Africa

^4^Institute of Human Virology, Abuja, Nigeria

^5^Erasmus Medical Center, University Medical Center Rotterdam, The Netherlands

^6^Population Council-Washington DC, USA

**Corresponding author**

Salisu Mohammed Ishaku

salisuishaku@yahoo.com

**Supplement I**: Comparative analyses of characteristics of completed versus loss to follow up at one year among women with hypertensive disorders in pregnancy and the normotensive counterpart.

|  | **HDP** | | **P Value** | **Normotensive** | | **P value** |
| --- | --- | --- | --- | --- | --- | --- |
| **Variables** | **Followed up n(mean)** | **Loss to follow up n(mean)** |  | **Followed up n(mean)** | **Loss to follow-up n(mean)** |  |
| Mean age | 278(29.8) | 132(29.1) | 0.641 | 58(28.2) | 20(29.0) | 0.557 |
| Mean parity | 278(3.9) | 132 (4.1) | 0.747 | 58 (4.2) | 20 (4.0) | 0.457 |
| Mean Gestational age at onset of HDP (SD) | 245(33) | 120(33.5) | 0.442 |  |  |  |
| Mean Gestational age at booking (SD) | 174(23.4) | 71(24.3) | 0.040 | 54(22.9) | 16(22.4) | 0.791 |
| Mean BMI at baseline | 271(29.2) | 121(28.2) | 0.958 | 58(28.2) | 19(26.2) | 0.235 |
| Mean systolic BP at baseline (mmHg) | 219(158.8) | 43(161.7) | 0.464 | 46(111.2) | 7(119.6) | 0.308 |
| Mean diastolic BP at baseline (mm/Hg) | 219(137.1) | 43(135) | 0.786 | 46(73.2) | 7(79.1) | 0.206 |
| Mean creatinine at baseline (mg/dL) | 278(0.949) | 126(1.191) | 0.014 | 58(0.848) | 19(0.742) | 0.135 |
| Cholesterol (mmol/l) | 277(4.529) | 127(4.052) | 0.008 | 58(4.296) | 19(4.573) | 0.454 |
| Triglycerides (mmol/l) | 276(1.798) | 122(1.823) | 0.819 | 54(1.631) | 19(1.458) | 0.303 |

**SUPPLEMENT II:** Distribution of Sociodemographic and Obstetric Characteristic of completed versus lost to follow up among the HPDs at one year

| **Variables** | **Followed up to 1 year** | **Loss to follow up at one year** |
| --- | --- | --- |
| Mean age (SD) | 29.8(6.2) | 29.1(19.9) |
| Mean Gestational age at onset of HDP (SD) | 33(9.9) | 33.5(7.3) |
| Mean Gestational age at booking (SD) | 23.4(6.8) | 24.3(5.6) |
|  |  |  |
| *Category of HDP* | | |
| Chronic Hypertension n (%) | 15(7.54) | 18(11.1) |
| Gestational hypertension n (%) | 52(26.1) | 20(12.4) |
| Pre-eclampsia n (%) | 107(53.8) | 92(56.8) |
| Eclampsia n (%) | 25(12.6) | 32(19.8) |
|  | | |
| Mean BMI at baseline (SD) | 29.2(7.9) | 28.2(7.5) |
| Mean BMI at 1 year (SD) | 28.7(7.2) | ___ |
| Mean systolic BP at baseline (SD) | 158.8(20.8) | 161.7(23.3) |
| Mean systolic BP at 1 year (SD) | 133.3(26.6) | ___ |
| Mean diastolic at baseline (SD) | 137.1(23.4) | 135(29.3) |
| Mean diastolic at 1 year (SD) | 86.1(18.3) | ___ |
